# Metabolic Syndrome Triggered by Fructose Diet Impairs Neuronal Function and Vascular Integrity in ApoE-KO Mouse Retinas: Implications of Autophagy Deficient Activation

**DOI:** 10.3389/fcell.2020.573987

**Published:** 2020-10-08

**Authors:** María C. Paz, Pablo F. Barcelona, Paula V. Subirada, Magali E. Ridano, Gustavo A. Chiabrando, Claudia Castro, María C. Sánchez

**Affiliations:** ^1^Departamento de Bioquímica Clínica, Facultad de Ciencias Químicas, Universidad Nacional de Córdoba, Córdoba, Argentina; ^2^Centro de Investigaciones en Bioquímica Clínica e Inmunología, Consejo Nacional de Investigaciones Científicas y Técnicas, Córdoba, Argentina; ^3^Instituto de Medicina y Biología Experimental de Cuyo, Consejo Nacional de Investigaciones Científicas y Técnicas, Facultad de Ciencias Médicas, Universidad Nacional de Cuyo, Mendoza, Argentina

**Keywords:** metabolic parameters, non-proliferative retinopathy, functional retinal changes, neurodegeneration, vascular permeability, cellular processes

## Abstract

Metabolic syndrome is a disorder characterized by a constellation of clinical findings such as elevated blood glucose, hyperinsulinemia, dyslipidemia, hypertension, and obesity. A positive correlation has been found between metabolic syndrome or its components and retinopathy, mainly at microvascular level, in patients without a history of diabetes. Here, we extend the investigations beyond the vascular component analyzing functional changes as well as neuronal and glial response in retinas of Apolipoprotein E knockout (ApoE-KO) mice fed with 10% w/v fructose diet. Given that autophagy dysfunction is implicated in retinal diseases related to hyperglycemia and dyslipidemia, the activation of this pathway was also analyzed. Two months of fructose intake triggered metabolic derangements in ApoE-KO mice characterized by dyslipidemia, hyperglycemia and hyperinsulinemia. An increased number of TUNEL positive cells, in addition to the ganglion cell layer, was observed in the inner nuclear layer in retina. Vascular permeability, evidenced by albumin–Evans blue leakage and extravasation of albumin was also detected. Furthermore, a significant decrease of the glial fibrillary acidic protein expression was confirmed by Western blot analysis. Absence of both Müller cell gliosis and pro-angiogenic response was also demonstrated. Finally, retinas of ApoE-KO FD mice showed defective autophagy activation as judged by LC3B mRNA and p62 protein levels correlating with the increased cell death. These results demonstrated that FD induced in ApoE-KO mice biochemical alterations compatible with metabolic syndrome associated with neuronal impairment and mild vascular alterations in the retina.

## Introduction

Metabolic Syndrome (MetS) is a complex disorder of metabolism considered a global epidemic that involves a high socio-economic cost (Sherling et al., 2017). This syndrome defined as a constellation of metabolic abnormalities including elevated blood glucose, hyperinsulinemia, dyslipidemia, hypertension and obesity (Schwartz and Reaven, 2006) affects approximately 20–25% of the adult population (Ibrahim et al., 2019). Individuals with MetS have two to threefold risk of developing cardiovascular disease and fivefold risk of developing type 2 Diabetes Mellitus (DM) (Wild et al., 2004). In this sense, deleterious effects of MetS extend beyond the heart affecting other organs such as the retina (Thierry et al., 2015; Zarei et al., 2017), doubling the number of people with visual impairment. Accumulating evidences, stemming from clinical and experimental research have shown that, in addition to the vascular component, neurons and glial cells are also affected in retinopathies and their dysfunction may contribute to disease progression (Dorrell et al., 2010; Liu et al., 2010; Fu et al., 2015). Although, type 2 DM is frequently associated with MetS, this syndrome can also occur as an isolated entity, which provides an opportunity to study the early retinal consequences of systemic metabolic changes. While diagnosis of DM has clear-cut definitions, many patients experience before the onset of frank diabetes a series of biochemical alterations which affect the cardiovascular and metabolic condition (Forouhi et al., 2007; Nathan et al., 2007), contributing the latter to development of retinopathy (Mbata et al., 2017). The association of MetS and retinopathy in patients without a history of diabetes has been researched, mainly at vascular level, in a number of studies (Wong et al., 2004; Keenan et al., 2009; Peng et al., 2010; Liu et al., 2015). However, little is known about the functional retinal changes, neuroglial response and molecular mechanisms involved. Even though numerous animal models of MetS have been established (Wong et al., 2016) further investigations of its deleterious consequences on retina are still necessary. In this study, we hypothesized that a combination of genetic condition plus carbohydrate-rich diet would be sufficient to induce MetS and retinal damage. Specifically, ApoE-KO mice spontaneously hypercholesterolemic were fed with a diet supplemented with 10% of fructose (FD), an important and pervasive sweetener in Western diets that produces impaired glucose tolerance and increases the blood triglyceride concentrations (Tappy and Rosset, 2019). In this *in vivo* model of MetS, we characterized the effects of metabolic derangement on retinal function over time and analyzed the vascular, neuronal and glial alterations. Finally, given that autophagy deregulation is implicated in many retinal pathologies related to hyperglycemia and dyslipidemia (Meng et al., 2017), we investigated if this cellular homeostatic mechanism (Kim et al., 2008; Rodriguez-Muela et al., 2012; Wen et al., 2019) was activated in FD-fed ApoE-KO mice, once retinal abnormalities were detected.

## Materials and Methods

### Mouse Model

**SCHEME 1:**
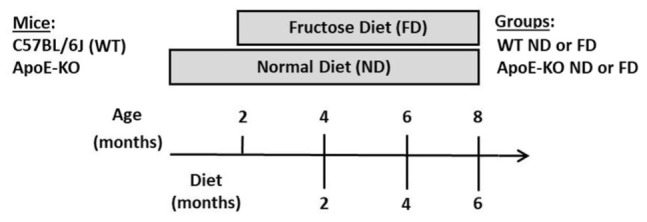
Experimental design showing the mice strain, their age (months), type of diets and study-time points (months) and the working-groups (WT ND, WT FD, ApoE-KO ND, and ApoE-KO FD).

Male C57BL/6J wild type (WT) and Apolipoprotein E-deficient (ApoE-KO) mice on C57BL/6J background (The Jackson Laboratories, Bar Harbor, ME, United States), 8 weeks of age (20–22 g), were used for this study. Mice were maintained under standard laboratory conditions of temperature (22 ± 1°C) and light (12-h light/12-h dark cycle) with free access to food and water. Mice were randomly divided in the following groups: WT mice fed either a normal diet (ND) with free access to tap water (WT ND) or fructose diet (WT FD) receiving 10% (w/v) D (−) fructose p.a. (Biopack, Argentina) in drinking water. Additional group of age-matched ApoE-KO mice were given a ND (ApoE-KO ND) or fructose diet (ApoE-KO FD). All experimental groups received a standard commercial mice chow. Fructose solution was replaced every 2 days. Mice were sacrificed at three time points: 2, 4, and 6 months of feeding (Scheme 1). Whole eyes or retinas of sacrificed mice were collected and processed for Western blot, Real-Time PCR (qRT-PCR), immunohistochemistry, immunofluorescence or flat-mount assays. In order to analyze the autophagy pathway, some mice received an intraperitoneal (i.p.) injection of 60 mg/kg chloroquine (CQ, Sigma-Aldrich, St. Louis, MO, United States) diluted in sterile phosphate buffered saline (PBS) 4 h before sacrifice. At least four mice per group were used for each condition depending on the experiment. Experimental procedures were designed and approved by the Institutional Animal Care and Use Committee (CICUAL) of the Faculty of Chemical Sciences, National University of Córdoba (Res. HCD 1199/17). All efforts were made to reduce the number of animals used.

### Blood Glucose Measurements

Plasma glucose levels were determined in tail vein blood samples, using the Free Style Optium blood glucose meter (Abbot Laboratories Argentina) in fasted (overnight, *n* = 4) or non-fasted mice (*n* = 8). The measurements were performed at the same time of day in each experimental condition.

### Intraperitoneal Glucose Tolerance Test

The i.p. glucose tolerance test (IPGTT) was performed at 4 months of feeding. Animals were fasted overnight before the experiments. For the IPGTT, mice received an i.p. 1 g/kg (body weight) of glucose injection. Blood samples for plasma glucose measurement were collected from the tail vein at time 0 (before glucose injection) and 30, 60, and 120 min after glucose administration (*n* = 4). The area under the curve (AUC) was calculated using the trapezoidal rule estimation by GraphPad Prism 7.0 software.

### Lipid Profile and Insulin Levels

At the end of the experimental period and prior euthanasia, triglycerides, high-density lipoprotein (HDL) cholesterol, low-density lipoprotein (LDL) cholesterol, total cholesterol and insulin were measured after overnight fasting, in heparinized blood samples collected from cardiac puncture in anesthetized animals via i.p. with ketamine (35 mg/kg)/xylazine (3.5 mg/kg). Different plasmatic cholesterol forms and triglyceride concentrations were determined using automatized commercial kits by enzymatic colorimetric methods (Roche, Buenos Aires, Argentina, *n* = 6 – 8). Insulin was measured by ELISA (Crystal Chem, United States, *n* = 4).

### Electroretinography (ERG)

Electroretinographic activity was recorded after 2, 4, and 6 months of feeding according to previously described procedures (Ridano et al., 2017; Lorenc et al., 2018; Subirada et al., 2019). Briefly, after overnight dark adaptation and under dim red illumination, mice were anesthetized with ketamine/xylazine (i.p.), the pupils were dilated with 1% tropicamide (Midryl, Alcon, Buenos Aires, Argentina) and the cornea was lubricated with gel drops of 0.4% polyethylene glycol 400 and 0.3% propylene glycol (Systane, Alcon, Buenos Aires, Argentina) to prevent damage. After body temperature stabilization on a 37°C warming pad for 10 min, mice were exposed to a light stimulus at a distance of 20 cm. A reference electrode was inserted on the back between the ears, a grounding electrode was attached to the tail, and a gold electrode was placed in contact with the central cornea. Electroretinograms (ERG) were simultaneously recorded from both eyes and ten responses to not dimmed flashes of white led light (5 cd.s/m2, 0.2 Hz) from a photic stimulator (light-emitting diodes) set at maximum brightness were amplified, filtered (1.5-Hz low-pass filter, 1000 high-pass filter, notch activated) and averaged (Akonic BIO-PC, Argentina).

The a-wave was measured as the difference in amplitude between the recording at onset and trough of the negative deflection, and the b-wave amplitude was measured from the trough of the a-wave to the peak of the b-wave. The latencies of the a- and b-waves were measured from the time of flash presentation to the trough of the a-wave or the peak of the b-wave, respectively. Responses were averaged across the two eyes for each mouse (*n* = 6 – 12).

Oscillatory potentials (OPs) were assessed as previously described (Moreno et al., 2005). Briefly, the same photic stimulator with a 0.2 Hz frequency and filters of high (300 Hz) or low (100 Hz) frequency were used. The OPs amplitude was estimated by measuring the heights from the baseline drawn between the troughs of successive wavelets to their peaks. The sum of three central peaks of OPs was used for statistical analysis. Responses were averaged across the two eyes for each mouse (*n* = 6 – 12).

### Vascular Permeability

Vascular permeability was analyzed by measuring albumin–Evans blue complex leakage from retinal vessels as previously described (Ma et al., 1997; Barcelona et al., 2016). Briefly, mice (*n* = 3) were injected intravenous (i.v.) through the vein of the tail with a solution of Evans blue (2% w/v dissolved in PBS). Immediately after injection, animals turned visibly blue, confirming the dye uptake and distribution. After 48 h, mice were transcardially perfused with saline solution and flat-mounted retinas were obtained. Microphotographs were taken using identical exposure time, brightness, and contrast settings (Fernandez et al., 2011).

### Labeling of Flat-Mount Retinas

Mice were euthanized at 4 months of diet and eyes were enucleated and fixed with freshly prepared 4% paraformaldehyde (PFA) for 2 h at room temperature. Corneas were removed with scissors along the limbus and the whole retinas were dissected. Then, they were blocked and permeabilized in Tris-buffered saline (TBS) containing 5% Bovine Serum Albumin (BSA, Sigma-Aldrich, United States) and 0.1% Triton-X-100 during 6 h at 4°C. After that, retinas were incubated overnight with Isolectin IB4 Alexa fluor-488 conjugated (GSA-IB4) from Molecular Probes, Inc. (1/100; Eugene, OR, United States) in combination with rabbit polyclonal Anti-Glial Fibrillary Acidic Protein (GFAP) antibody from Dako (1/200; Carpinteria, CA, United States) or mouse polyclonal alpha Smooth Muscle Actin (α-SMA) antibody from Dako (1/100; Carpinteria, CA, United States). Then, retinas were washed with TBS 0.1% Triton-X-100 and incubated with secondary antibodies including goat against rabbit or mouse IgG conjugated with Alexa Fluor 488 and 594 (1/250; Molecular Probes, Eugene, OR, United States) during 1 h at room temperature. Retinas were then washed with TBS containing 0.1% Triton-X-100, stored in PBS at 4°C and examined by confocal laser-scanning microscopy (Olympus FluoView FV1200; Olympus Corp., New York, NY, United States). Each image is the flatten result of 10 photos (10 μm) taken at plane z. Vascular density, vascular diameter and branching of the vasculature of the different experimental groups were quantified from GSA-IB4 immunostained flats, three different flats of each experimental group were average (*n* = 3). The vascular density was quantified as fluorescence intensity labeling (% area), using a square of 200 mm^2^ (see scheme of quantification in Figure 3B) which was positioned in nine different places in each microphotograph, using Image J Fiji software (National Institutes of Health, Bethesda, MD, United States). The vascular diameter was quantified as the average (pixels) of three measurements in different places of three big arterioles in each microphotograph. Three areas of quantification were defined by using concentric circles around the optic nerve separated by 200 μm (see scheme of quantification in Figure 3B). Representative image and quantification at only one height is shown as no significant difference was observed at any height measured. The branching was quantified counting the primary branches from a big arteriole, since the optic nerve to the periphery. α-SMA immunostaining was quantified as fluorescence intensity labeling (% area) from four different flat-mounts of each experimental group (*n* = 4) using Image J Fiji software (National Institutes of Health, Bethesda, MD, United States).

### Retinal Cryosection, Protein Extract and RNA Sample Preparation

For cryosection, eyes after 4 months of feeding were enucleated, fixed during 2 h with 4% PFA at room temperature, and incubated overnight in 10, 20, and 30% of sucrose in PBS at 4°C. Then, they were embedded in optimum cutting temperature (OCT, Tissue-TEK, Sakura) compound, and 10-μm-thick radial sections were obtained by using a cryostat, according to general methods (Subirada et al., 2019). Retinal cryosections were then stored at −20°C under dry conditions until immunohistochemical analysis. Neural retinas were dissected from Retinal Pigmentary Epithelium/choroid layers for Western blot and qRT-PCR analysis (Ridano et al., 2017; Lorenc et al., 2018). Protein extracts were obtained from retinas after homogenization with a lysis buffer containing 20 mM Tris-HCl pH 7.5, 137 mM NaCl, 2 mM EDTA pH 8, 1% Nonidet P40, 1 mM phenylmethylsulfonyl fluoride (PMSF), 2 mM sodium ortovanadate and protease inhibitor cocktail (Sigma Aldrich, St. Louis, MO, United States), and were sonicated during 20 s at 40% amplitude. In addition, some neural retinas were disrupted in 500 μL Trizol (Invitrogen) and were stored at −80°C until RNA extraction.

### Immunofluorescence

Immunostaining was performed as described previously (Ridano et al., 2017; Lorenc et al., 2018; Subirada et al., 2019). Briefly, mouse cryosections were washed in PBS, blocked with 2% of BSA in PBS containing 0.1% Tween 20, for 1 h and then incubated overnight at 4°C with the following primary antibodies: rabbit polyclonal Anti-GFAP antibody from Dako (1/100; Carpinteria, CA, United States), rabbit polyclonal anti-LC3B (1/100; L7543, Sigma Aldrich) and mouse monoclonal anti-p62 (1/100; ab56416, Abcam). Then, sections were washed with 0.1% Tween 20 in PBS and incubated with secondary antibodies including goat against rabbit or mouse IgG conjugated with Alexa Fluor 488 or 594 (1/250; Molecular Probes, Eugene, OR, USA) during 1 h at room temperature. The sections were also counterstained with Hoechst 33258 (1:3000; Molecular Probes) for 7 min. After a thorough rinse, the sections were mounted with Fluor Save (Calbiochem, La Jolla, CA, United States) and cover slipped. The labeling was visualized using a confocal laser-scanning microscope (Olympus Fluo View FV300 or FV1200; Olympus Corp., New York, NY, United States). Finally, images from three different retinas from each experimental group (*n* = 3), were processed with Image J software (National Institutes of Health, Bethesda, MD, United States). Negative controls without incubation with primary antibody were carried out to detect unspecific staining (data not shown). Negative controls without incubation with primary antibody were carried out to detect unspecific staining (data not shown). Vesicle quantification: in each cryosection, two images were acquired at the central area, next to the optic nerve head. Images were taken with oil 40X objective in the best confocal resolution condition. A total of 10 consecutive images were acquired in z plane, with a step size of 1 μm. The quantification of the number of LC3B positive vesicles was carried out in deconvolved images with ImageJ FIJI software, analyze particles plugin. The measurement of the number of puncta was done in a standard ROI (20 μm × 20 μm) area and further correlated to the number of nuclei present in the ROI. At least 5 ROIs were quantified from each layer of the retina in each slide for every condition.

### Western Blot

Protein concentration of retinal extracts were determined by a Bicinchoninic Acid Protein Assay Kit (Pierce BCA, Thermo Scientific, United States) and 10 – 20 μg of proteins were electrophoresed in 10 or 15% sodium dodecyl sulphate polyacrylamide gel electrophoresis (SDS-PAGE). After electrophoresis, proteins were transferred to nitrocellulose membranes (Amersham Hybond ECL; GE Healthcare Bio-Sciences AB, Uppsala, Sweden). To prevent non-specific binding, membranes were blocked with 5% BSA in TBS containing 0.1% Tween-20 (TBST) during at least 1 h at room temperature. Then, blots were incubated with primary antibodies diluted in TBST or 1% BSA in TBST for 1 h at room temperature or overnight at 4°C, according to the antibody. The following primary antibodies were used: rabbit polyclonal anti-GFAP (1/1000; Dako, Carpinteria, CA, United States, *n* = 4), mouse monoclonal anti- glutamine synthetase (GS) (1/500; Millipore Corporation, MA, United States, *n* = 5), goat polyclonal anti-serum albumin (1/5000; Abcam, Argentina, n = 3), rabbit anti-LC3B (1/1000; Sigma-Aldrich, United States, *n* = 3), mouse monoclonal anti SQSTM1/p62 (1/1000; Abcam, *n* = 3) and loading proteins such as, mouse monoclonal anti-β actin (1/2000; Sigma-Aldrich, United States) or mouse monoclonal anti-α tubulin (1/2000; Sigma-Aldrich). Blots were incubated with IRDye 800 CW or IRDye 695 CW donkey anti-rabbit IgG, IRDye 800 CW or IRDye 695 CW donkey anti-mouse IgG or IRDye 695 CW donkey anti-goat IgG antibodies (1/15000 in TBS with 1% BSA) for 1 h, protected from light. After washing with TBST, membranes were visualized and quantified using the Odyssey Infrared Imaging System (LI-COR, Inc., Lincoln, NE, United States). All the assays were performed in triplicate and results are representative of at least three independent experiments (*n* = 3 – 5).

### qRT-PCR

Total RNA was extracted from neural retinas using Trizol (Invitrogen), according to the manufacturer’s instructions and was processed as previously reported (Ridano et al., 2017; Subirada et al., 2019). Briefly, 1 μg of total RNA was reverse-transcribed in a total volume of 20 μL using random primers (Invitrogen, Buenos Aires, Argentina) and 50 U of M-MLV reverse transcriptase (Promega Corp.). For qPCR, cDNA was mixed with 1x SYBR Green PCR Master Mix (Applied Biosystems) and the forward and reverse primers (LC3B forward: CGC TTG CAG CTC AAT GCT AAC/LC3B reverse: CTC GTA CAC TTC GGA GAT GGG, Hypoxia-inducible factor-1α (HIF-1α) forward: CACCGATTCGCCATGGA/HIF-1α reverse: TTCGACGTTCAGAACTCATCTTTT, Vascular endothelial growth factor (VEGF) forward: AGAGCAGAAGTCCCATGAAGTGA/VEGF reverse: TCAATCGGACGGCAGTAGCT, Tumoral necrosis factor-α (TNF-α) forward: AGCCGATG GGTTGTACCTTGTCTA/, TNF-α reverse: TGAGAT AGCAAATCGGCTGACGGT and Interleukin-6 (IL-6) forward: ATCCAGTTGCCTTCTTGGGACTGA/IL-6 reverse: TAAGCCTCCGACTTGTGAAGTGGT), were carried out on an Applied Biosystems 7500 Real- Time PCR System with Sequence Detection Software v1.4. The cycling conditions included a hot start at 95°C for 10 min, followed by 40 cycles at 95°C for 15 s and 60°C for 1 min. Specificity was verified by melting curve analysis. Results were normalized to β-actin forward: GGCTGTATTCCCCTCCATCG/β-actin reverse: CCAGTTGGTAACAATGCCATGT. Relative gene expression was calculated according to the 2-ΔΔCt method. Each sample was analyzed in triplicate. No amplification was observed using as template water or RNA samples incubated without reverse transcriptase during the cDNA synthesis (data not shown). All the assays were performed in triplicate and results are representative of at least three independent experiments (*n* = 3 – 4).

### TUNEL Assay

Cell death was examined by terminal deoxynucleotidyltransferase biotin dUTP nick end labeling (TUNEL) assay (Roche, Mannheim, Germany) following the manufacturer’s instructions. The peroxidase label was detected with diaminobenzidine hydrochloride (Sigma-Aldrich, United States). Slides were counterstained with methyl green to visualize retinal layers and then mounted with DPX Mounting Media (Sigma-Aldrich, United States). Negative controls without enzyme were processed in order to avoid false positive results (data not shown). For each section, TUNEL-positive nuclei staining brown were counted (nucleus/mm) from three randomly selected fields on either side of the optic nerve and the average values from three slices/eye were combined to produce a mean value of each mouse (*n* = 3). Images were obtained under a light microscope (Nikon Eclipse TE2000-E, Japan).

### Statistical Analysis

Statistical analysis was performed using the GraphPad Prism 7.0 software. A *p*-value < 0.05 was considered statistically significant. Parametric or non-parametric tests were used according to variance homogeneity evaluated by *F* or Barlett’s tests. Kruskal–Wallis followed by Dunn’s multiple comparisons post-test or two-way ANOVA followed by Bonferroni multiple comparisons post-test. Data represent the mean ± standard error of the mean (SEM) or the median with the interquartile range depending on parametric or non-parametric test.

## Results

### Fructose Diet Triggered MetS in ApoE-KO Mice

**FIGURE 1:**
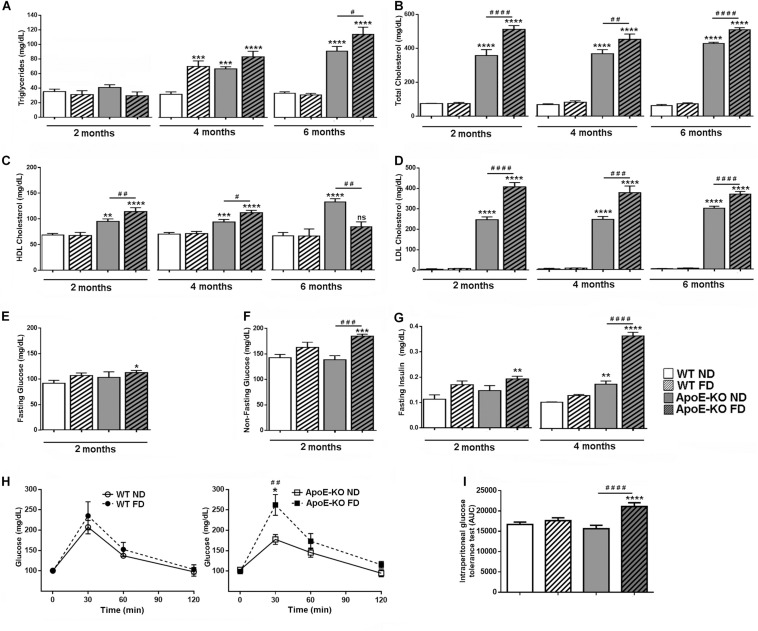
Metabolic profile of peripheral blood in WT and ApoE-KO mice fed with ND or FD during 2, 4, or 6 months. **(A)** Triglyceride levels (mg/dl), *n* = 6 – 8. **(B)** Total cholesterol levels (mg/dl), *n* = 6 – 8. **(C)** HDL-cholesterol levels (mg/dl), *n* = 6 – 8. **(D)** LDL-cholesterol levels (mg/dl), *n* = 6 – 8. **(E)** Glycemic levels (mg/dl) in fasted mice (*n* = 4). **(F)** Glycemic levels (mg/dl) in non-fasted mice (*n* = 8). **(G)** Insulin levels (mg/dl) in fasted mice (*n* = 4). **(H)** Glycemic levels (mg/dl) before (time 0) and 30, 60, and 120 min after glucose injection (1 g/Kg, i.p.) of the different experimental groups at 4 months of diet (all experimental groups were statistically analyzed together but were shown in two graphs for better visualization, *n* = 4) **(I)** IPGTT, bars show the AUC quantification of glycemic levels of graph **(H)**. Data correspond to mean ± SEM. Two-way ANOVA followed by Bonferroni *post hoc* test. Not significant (ns), *p < 0.05, **p < 0.01, ***p < 0.001, and ****p < 0.0001 vs. WT ND; ^#^*p* < 0.05, ^##^*p* < 0.01, ^###^*p* < 0.001, and ^####^*p* < 0.0001 ApoE-KO FD vs. ApoE-KO ND.

Representative biochemical parameters of plasma lipid profile were quantified in WT mice and ApoE-KO mice fed with ND or FD at 2, 4, and 6 months. Triglyceride levels were similar in all experimental groups at 2 months of diet (Figure 1A). However, at 4 months significant differences were observed in WT mice fed with FD (*p* < 0.001) and ApoE-KO mice with either ND (*p* < 0.001) or FD (*p* < 0.0001) compared with WT ND group (Figure 1A). Interestingly, at 6 months of diet, an additional increase was observed for triglycerides in ApoE-KO FD (*p* < 0.05) compared with ApoE-KO ND group, whereas in WT mice fed with FD group it returned to baseline levels (Figure 1A). Regarding the total cholesterol levels, a clearly significant difference was observed between ApoE-KO and WT mice, independently of the diet, at every evaluated time point (Figure 1B, *p* < 0.0001). Notably, total cholesterol in the ApoE-KO FD group was significantly higher than ApoE-KO ND group (Figure 1B, *p* < 0.0001). Plasma HDL cholesterol significantly increased in hypercholesterolemic mice (*p* < 0.001) compared to WT mice at 2 and 4 months regardless of the diet, and they began to decline at 6 months in ApoE-KO FD group (Figure 1C). In addition, a significant increase in the LDL cholesterol level was observed throughout the period evaluated in ApoE-KO mice (*p* < 0.0001) compared to WT mice, and this rise was higher in ApoE-KO FD group (*p* < 0.0001) compared with ApoE-KO ND group (Figure 1D).

In a previous study, we reported an increased concentration of plasma glucose in ApoE-KO mice fed with FD at 2 months of diet (Cannizzo et al., 2012), which was confirmed in this study under fasting and non-fasting conditions. A significant increase in the fasting glucose levels was observed in ApoE-KO FD group as compared with WT ND group (Figure 1E, *p* < 0.05), whereas in non-fasting conditions, it was shown an additional significant difference between ApoE-KO groups (Figure 1F, *p* < 0.001). Fasted insulin was higher in ApoE-KO FD group (*p* < 0.01) compared with WT ND mice at 2 months of diet. At 4 months, insulinemia was higher in ApoE-KO ND group (*p* < 0.01) and also in ApoE-KO FD group (*p* < 0.0001), showing an additional rise in ApoE-KO FD group (*p* < 0.0001) compared with ApoE-KO ND group (Figure 1G). Finally, in order to evaluate the ability of the mice to metabolize glucose, the IPGTT was performed in all groups of animals at 4 months of diet. Glucose levels assessed before and 30, 60, and 120 min after an i.p. administration of 1 g/kg of glucose, showed a significant increase in ApoE-KO FD group compared with ApoE-KO ND mice after 30 min of glucose injection (Figure 1H, *p* < 0.01). The average of AUC is showed in Figure 1I. Higher values of AUC were observed for the ApoE-KO FD group (*p* < 0.0001) when compared with ApoE-KO ND group whereas no differences were observed between WT ND and FD groups (Figure 1I).

Taken together, these results demonstrated that 2 months of fructose intake triggered metabolic derangements in spontaneously hypercholesterolemic ApoE-KO mice, which mimics human MetS characterized by dyslipidemia, high insulin levels and hyperglycemia.

### Fructose Diet Promoted Neuronal Impairment in the Inner Nuclear Layer of ApoE-KO Mice Retinas

**FIGURE 2:**
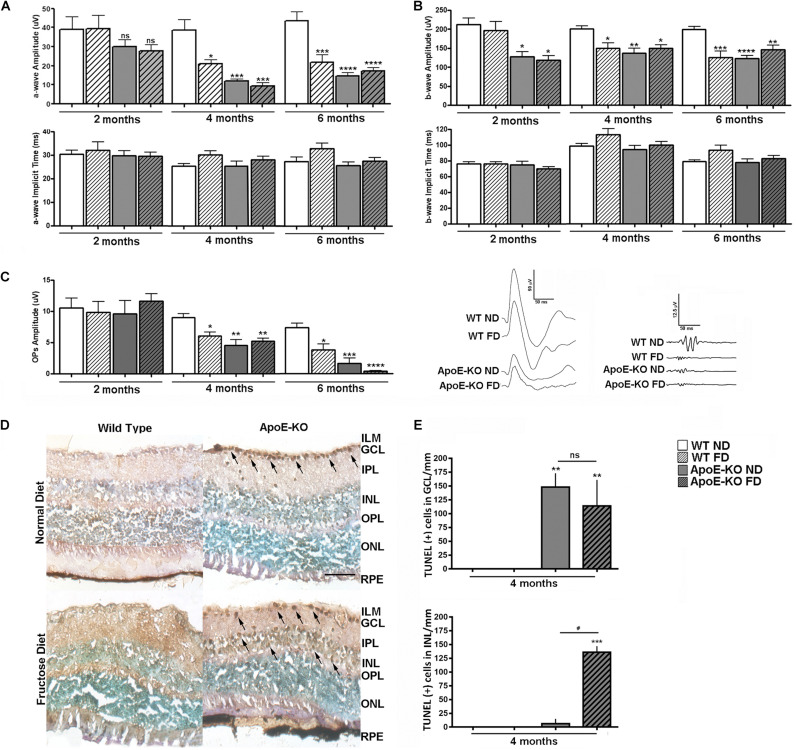
Scotopic ERG after white led flash (5 cd.s/m2, 0.2 Hz) in WT and ApoE-KO mice fed with ND or FD during 2, 4, or 6 months (*n* = 6 – 12). **(A)** Bars represent the average of the amplitude (top panel) and implicit time (bottom panel) of a wave (ms) of the different experimental groups. **(B)** Bars represent the average of the amplitude (top panel) and implicit time (bottom panel) of b wave (ms) of the different experimental groups. **(C)** Bars represent the average of the OPs amplitudes, obtained by sum of OPs 2, OPs 3, and OPs 4, of the different experimental groups. The panel show representative registers of mixed response (scale 50 uV/50 ms) and OPs (scale 12.5 uV/50 ms) at 4 months of diet. **(D)** Representative photomicrographs of cell death analysis by TUNEL/DAB and methyl green counterstaining, in retinal cryosections (10 μm) of the different experimental groups at 4 months of diet (*n* = 3). Cell death was observed as TUNEL positive cells (arrows) in GCL of ApoE-KO (ND and FD) mice and in INL of ApoE-KO FD mice, scale bar: 50 μm, 400x magnification. **(E)** Bars represent the average number of TUNEL positive cells/mm in GCL and INL of retinas of the different experimental groups at 4 months of diet. Data correspond to mean ± SEM (*n* = 4 – 14, depending on the experiment). Two-way ANOVA followed by Bonferroni *post hoc* test or Kruskal–Wallis followed by Dunn’s *post hoc* test. Not significant (ns), *p < 0.05, **p < 0.01, ***p < 0.001, and ****p < 0.0001 vs. WT ND; ns, ^#^*p* < 0.05 ApoE-KO FD vs. ApoE-KO ND. ILM, inner limiting membrane; GCL, ganglion cell layer; IPL, inner plexiform layer; INL, inner nuclear layer; OPL, outer plexiform layer; ONL, outer nuclear layer; RPE, retinal pigmentary epithelium.

Next, we analyzed the functional status of the retinas by scotopic ERG at 2, 4, and 6 months of diet. The a-wave amplitude was lower, although not statistically significant, in both ApoE-KO (ND, *p* < 0.9999 and FD, *p* = 0.9865) groups at 2 months of diet respect to WT ND group, differing significantly at 4 months even for the WT DF group (*p* < 0.05) (Figure 2A). These ERG changes were maintained at 6 months of diet. Regarding the a-wave implicit time, no changes were observed among the experimental groups at every evaluated time points (Figure 2A). At 2 months of diet, the b-wave amplitude and -implicit time values showed a similar profile as the a-wave, excepting that both ApoE-KO (ND and FD) groups evidenced an early reduction in b-wave amplitude (*p* < 0.05) (Figure 2B). This effect was maintained at 4 and 6 months of diet in WT FD mice as well as in both ApoE-KO mice groups whereas the b-implicit time did not show changes. Regarding the sum of OPs amplitude, a progressive decrease in the ApoE-KO groups (ND and FD) as well as in WT FD respect to WT ND group was observed at 4 months of diet. This reduction was more pronounced in the ApoE-KO mice at 6 months of FD (*p* < 0.0001) (Figure 2C). Representative scotopic ERG traces at 4 months of diet are also shown in Figure 2. In line with these results, TUNEL- positive cell nuclei were detected in retinas of ApoE-KO mice in either ND or FD at 4 months of diet (Figure 2D). Quantitative analysis showed a significant increase in the number of TUNEL positive cells in ganglion cell layer (GCL) in both ApoE-KO groups (*p* < 0.01) while an additional increase in the inner nuclear layer (INL; mainly neurons such as bipolar and amacrine cells), was observed in the ApoE-KO FD group (*p* < 0.001) (Figure 2E). The number of TUNEL positive cells showed no significant difference in INL and GCL between ApoE-KO groups at 6 months of FD (data not shown). Overall, these results reflected an early deterioration in retinal function and cell viability in ApoE-KO mice, which was intensified with the progressive fructose intake.

### Fructose Diet Induced Vascular Permeability and Astrocyte Impairment in ApoE-KO Mice

**FIGURE 3:**
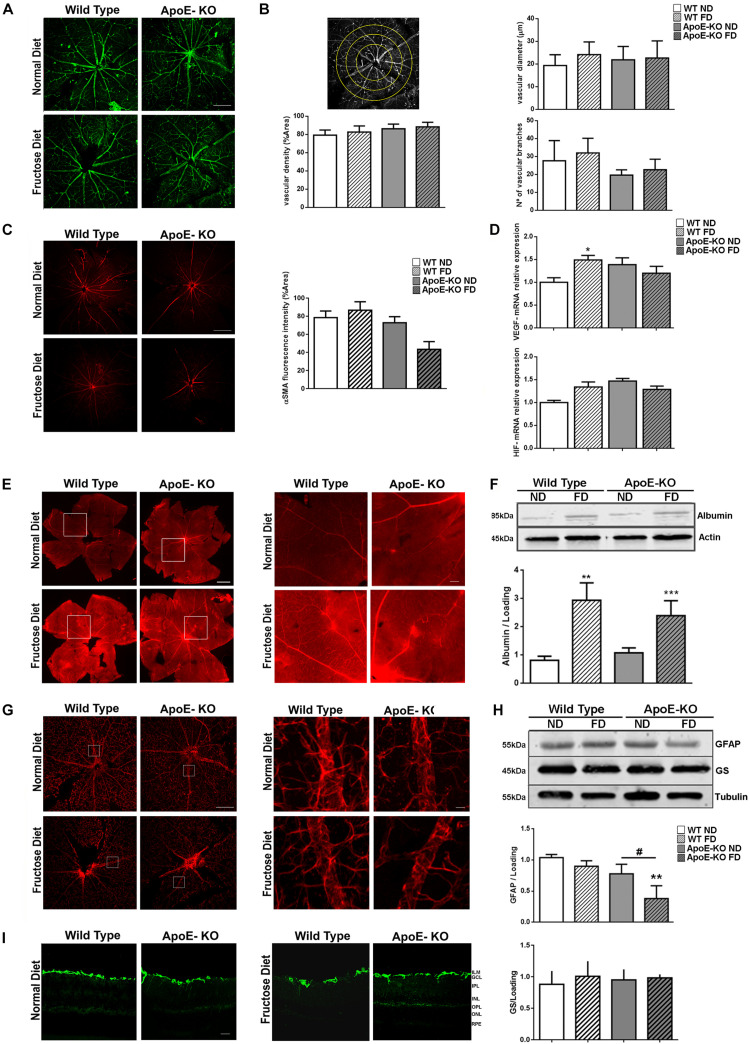
Vascular analysis. **(A)** Representative photomicrographs (scale of 100 μm, 100x magnification) of GSA-IB4 labeled (green) blood vessels in flat-mounts of the central area of whole retinas in the different experimental groups at 4 months of diet (n = 3). **(B)** Scheme of quantification (top left panel), bars represent the average vascular density (% area, bottom left panel), the average vascular diameter (μm, top right panel) and the average vascular branching (bottom right panel) of flat-mounted whole retinas of mice from the different experimental groups at 4 months of diet. **(C)** representative photomicrographs (scale 100 μm, 50x magnification) of α-SMA labeled (red) blood vessels in flat-mounts of the central area of whole retinas of mice from the different experimental groups at 4 months of diet (*n* = 4). Bars represent the average vascular density (% area). **(D)** Bars represent the average VEGF (top panel) and HIF-1α (bottom panel) mRNA levels relative to actin in mouse retinal extracts of the different experimental groups at 4 months of diet (*n* = 4). **(E)** Representative photomicrographs (scale 500 μm, 100x magnification) of albumin–Evans blue-complex leakage (red) in flat-mounts of the whole retinas in mice from the different experimental groups at 4 months of diet (*n* = 3). Square indicate the place of 1000x magnification, scale 250 μm. **(F)** Bars represent albumin protein relative to actin levels in retinal extracts of mice from the different experimental groups at 4 months of diet (bottom panel, *n* = 3). Representative blot is showed (top panel). **(G)** representative photomicrographs (scale of 100 μm, 50x magnification) of GFAP labeled (red) in flat-mounted of central area of the whole retinas of mice from the different experimental groups at 4 months of diet (*n* = 3). Squares indicate the place of 500x magnification, scale 500 μm. **(H)** Bars represent average protein expression of GFAP (*n* = 4) and GS (*n* = 5) relative to tubulin in retinal extracts of mice from the different experimental groups at 4 months of diet (bottom panel). Representative blot is showed (top panel). **(I)** Representative photomicrographs (scale 25 μm, 200x magnification) of GFAP label (green) in retinal criosections (10 μm) of mice from the different experimental groups at 4 months of diet (*n* = 3). Data correspond to mean ± SEM. Two-way ANOVA followed by Bonferroni *post hoc* test or Kruskal–Wallis followed by Dunn’s *post hoc* test. *p < 0.05, **p < 0.01, and ***p < 0.001 vs. WT ND, ^#^*p* < 0.05 ApoE-KO FD vs. ApoE-KO ND. GCL, ganglion cell layer; IPL, inner plexiform layer; INL, inner nuclear layer; OPL, outer plexiform layer; ONL, outer nuclear layer.

Examination of GSA-IB4-labeled blood vessels in flat-mounted whole retinas was carried out at 4 months of FD. It showed a clear demarcation into large retinal vessels (arteries and veins) and retinal capillaries (Figure 3A). Quantitative measurements of major arteriolar blood vessel diameter showed a similar mean value for all experimental groups. In addition, the closely meshed microvasculature did not show any differences in density among groups. Similarly, blood vessels showed a regular branching aspect (Figure 3B). Next, we stained retinal flat-mounts with an antibody against α-SMA to analyze coverage of retinal blood vessels with smooth muscle cells. Interestingly, a loss of α-SMA positive cells was mainly observed in retinas of ApoE-KO mice after 4 months of FD (Figure 3C, *p* = 0,075). Then, we tested VEGF gene expression and its upstream factor, HIF-1α, as markers of the pro-angiogenic response in retinal extracts. Whereas HIF-1α mRNA levels did not differ among groups (Figure 3D), a slight but significant increase in VEGF mRNA level was observed in WT FD mice in comparison with retinas from the WT ND group (*p* < 0.05) (Figure 3D). Nevertheless, neither HIF-1α nor VEGF mRNA levels were modified in ApoE-KO (ND and FD) groups.

Next, we evaluated the Blood-Retinal Barrier (BRB) permeability in flat-mounted retinas by the albumin–Evans blue-complex leakage technique. In WT and ApoE-KO ND groups, the dye was exclusively observed within the vessel lumen of the retinal vasculature, with very low background fluorescence levels. However, focal sites of leakage were observed in WT and ApoE-KO mice fed with FD, with the dye diffusely distributed through the retinal parenchyma (Figure 3E). A common approach to quantify alterations in retinal vascular permeability is to determine retinal serum albumin as an endogenous marker for vascular leakage (Vinores et al., 1989; Vinores et al., 1990). We found increased levels of albumin in retinal extracts of WT (*p* < 0.01) and ApoE-KO (*p* < 0.001) mice fed 4 months with FD by Western blot assay (Figure 3F).

Considering these results, then we investigated astrocyte integrity by analyzing GFAP immunoreactivity in flat-mounted retinas at 4 month of FD. As shown in Figure 3G, in WT ND and FD as well as in ApoE-KO ND retinas, an intense GFAP immunoreactivity was observed in star-shaped astrocytes adjacent to the inner limiting membrane (Figure 3G). Western blot analysis confirmed the results seen by immunofluorescence and the quantification revealed a significant decrease of the GFAP protein expression in the ApoE-KO FD group (*p* < 0.01). Levels of GS, a glutamate detoxification enzyme, showed no changes among groups (Figure 3H). Moreover, it was demonstrated by GFAP staining on retinal cryosections that Müller glial cells (MGCs) remained not activated among groups (Figure 3I). In line with this result, no changes were seen in mRNA expression of IL-6 and TNF-α in retinal extracts, neither in WT and ApoE-KO FD mice nor in ApoE-KO ND group compared with WT ND group, after 4 months of diet (data not shown). Together, these results demonstrated that FD induced vascular permeability and astrocyte impairment without disclosing an outstanding participation of pro-angiogenic and pro*-* inflammatory pathways in ApoE-KO mice.

### Fructose Diet Induced Autophagy Deficient Activation in Retinas of ApoE-KO Mice

**FIGURE 4:**
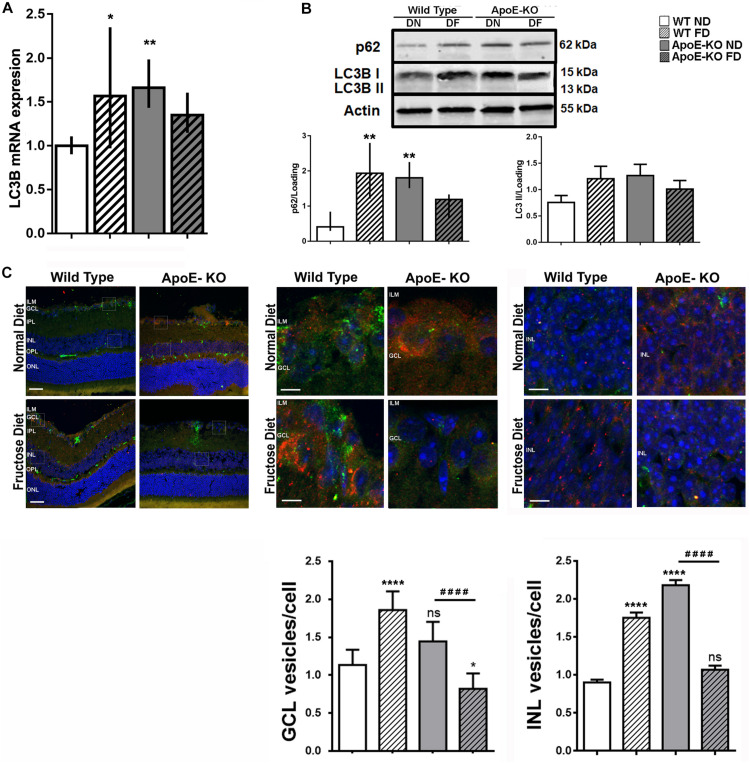
Autophagy pathway evaluation. **(A)** Bars represent the average LC3B mRNA levels relative to actin in mice retinal extracts from the different experimental groups at 4 months of diet. **(B)** Bars represent average protein expression of LC3B I (15 kDa), LC3B II (13 kDa) and p62 relative to actin, in retinal extracts of mice from the different experimental groups at 4 months of diet and 4 h after an i.p. CQ injection (bottom panel). Representative protein blot is showed (top panel). **(C)** Representative photomicrographs (scale 25 μm, 200x magnification) of LC3B label (red) and p62 label (green) in retinal cryosections (10 μm) of mice of the different experimental groups at 4 months of diet, 4 h after an i.p. CQ injection (top left panel). Squares indicate the place of 2000x magnification of the GCL (top central panel) and INL (top right panel); scale 250 μm. Bars represent the quantification of the average of LC3B positive vesicles/cell in GCL (bottom left panel) and in INL (bottom right panel). Data correspond to mean ± SEM (*n* = 3). Two-way ANOVA followed by Bonferroni *post hoc* test or Kruskal–Wallis followed by Dunn’s *post hoc* test. Not significant (ns), *p < 0.05, **p < 0.01, and ***p < 0.0001 vs. WT ND; ^####^*p* < 0.0001 ApoE-KO FD vs. ApoE-KO ND. ILM, inner limiting membrane; GCL, ganglion cell layer; IPL, inner plexiform layer; INL, inner nuclear layer; OPL, outer plexiform layer; ONL, outer nuclear layer; RPE, retinal pigmentary epithelium.

Recently, autophagy has demonstrated an important role in the eye diseases such as retinopathies as either a pro-survival or pro-death mechanism (Arroba et al., 2016; Boya et al., 2016; Cammalleri et al., 2016; Lopes de Faria et al., 2016; Dehdashtian et al., 2018; Subirada et al., 2019). In this sense, changes in the autophagy flux have been described in conditions of hyperglycemia and hyperlipidemia (Meng et al., 2017). On that basis, our aim was to determine if this catabolic process was modified early in retinas of ApoE-KO FD mice. For this purpose, samples were obtained at 4 months of diet, period at which changes in the retinal function were detected by ERG assay. Increased levels of LC3B mRNA in retinal extracts of WT FD (*p* < 0.05) and ApoE-KO ND (*p* < 0.01) mice respect to WT ND group were demonstrated by qRT-PCR assay. In ApoE-KO FD the levels of LC3B mRNA were similar to WT ND group (Figure 4A), suggesting a minor synthesis of proteins involved in the maintenance of the normal autophagy mechanism. Thus, the autophagy pathway was evaluated. For these experiments, we used an i.p. injection of CQ to monitor autophagosome accumulation by lysosomal blockade. Western blot assays showed that while p62/SQSTM1, a selective substrate of autophagy, did not change in ApoE-KO FD mice, an upregulation was observed in WT FD (*p* < 0.01) and ApoE-KO ND mice (*p* < 0.01). A similar trend was seen for protein levels of LC3BII, where this difference did not reach statistical significance (Figure 4B), correlating with the qRT-PCR data. Then, we focused on analysis of the number of autophagosomes located in GCL and INL where we had previously detected TUNEL positive cells and related ERG abnormalities. Quantitative analysis showed an increase in LC3B puncta in the GCL in WT FD (*p* < 0.0001) and a trend in ApoE-KO ND (*p* = 0.0597) while the ApoE-KO FD mice group showed an statistically reduction in LC3B puncta compared with WT ND (*p* < 0.05) or ApoE-KO ND mice (*p* < 0.0001) (Figure 4C). Regarding the INL, a similar increase was observed in WT FD (*p* < 0.0001) and ApoE-KO ND (*p* < 0.0001) groups whereas no changes were observed in ApoE-KO at 4 months of FD respect to WT ND group (Figure 4C).

These findings clearly showed defective autophagy pathway activation in retinas from ApoE-KO mice fed with FD, correlating with retinal dysfunction observed at 4 months of diet.

## Discussion

In this study, WT and ApoE-KO mice were used to investigate the effect of metabolic alterations on retinal tissue induced by fructose feeding. These studies included functional retinal analysis over time as well as vascular, glial and neuronal response. Our results showed that WT mice fed with FD as well as ApoE-KO ND mice evidenced some metabolic alterations that were correlated with a mild retinal damage. However, only ApoE-KO mice fed with fructose exhibited hallmark features of MetS, associated with neuronal impairment and mild vascular alterations in the retina.

ApoE plays a critical role in lipoprotein metabolism contributing to the removal of chylomicrons, VLDL and IDL remnants by its interaction with the specific high affinity hepatic ApoE receptor as well as with the hepatic LDL receptor (Plump et al., 1992). Thus, ApoE deficiency causes the inability to eliminate these lipoprotein remnants and consequently increases total cholesterol and triglycerides in circulation. Our findings indicated that 2 months of fructose intake, triggered metabolic derangements in spontaneously hypercholesterolemic ApoE-KO mice, mimicking MetS characterized by dyslipidemia, high insulin levels and hyperglycemia. Total cholesterol levels showed a significant increase at 2 months of FD in ApoE-KO mice differentiating from both WT groups and even from ApoE-KO ND group, which was maintained throughout the evaluated time points. A similar profile was obtained with LDL cholesterol whereas the HDL cholesterol level in ApoE-KO FD group was similar to WT ND at 6 months of FD. Therefore, a decrease in the HDL/LDL cholesterol ratio was observed in ApoE-KO FD mice. Regarding the triglycerides levels, an increase was observed in both ApoE-KO mice groups since 4 months of diet becoming remarkably different with the fructose ingestion at 6 months respect to ApoE-KO ND. It is of interest to note that plasmatic levels of triglycerides increased in WT mice at 4 months of FD. Observations from numerous mechanistic studies in cells, animals, healthy volunteers, and patients clearly demonstrated that fructose has lipogenic potential. Hepatic fructose metabolism rapidly produces gluconeogenesis and precursors for lipogenesis, while intermediary fructose metabolites also act as nutritional regulators of the major transcription factors that control these pathways. Accordingly, fructose ingestion increases plasma triglyceride levels (Chong et al., 2007; Parks et al., 2008; Teff et al., 2009). However, decreased levels of triglycerides observed in WT FD mice at 6 months suggested a hepatic homeostatic compensation.

The combination of genetic condition and carbohydrate-rich diet mimicked metabolic alterations experienced by individuals before the onset of frank diabetes such as fasting and non-fasting hyperglycemia, which were accompanied by hyperinsulinemia and glucose intolerance (Nathan et al., 2007; Mbata et al., 2017). These metabolic features compatible with MetS in humans were accurately reproduced in ApoE-KO FD, but not in the other experimental groups. Thus, altered lipid and carbohydrate profile associated with hyperinsulinemia clearly highlights to ApoE-KO FD mice as a novel animal model with characteristics of human MetS.

It is known that abnormal visual and retinal function usually precedes morphological alterations that characterize retinopathies in humans (Juen and Kieselbach, 1990; Gardner et al., 1997; Verma et al., 2009). The function of the neural components of the INL and the outer nuclear layer (ONL), assessed by ERG, is usually altered prior to the development of fundoscopically evident retinopathy changes. The most common ERG abnormality observed in these patients is a reduction in the amplitude and/or an increase in implicit time of OPs (Holopigian et al., 1992; Wachtmeister, 1998; Harrison et al., 2011; Bronson-Castain et al., 2012). Concerning the ERG results, most of the functional changes observed at 2 and 4 months of diet mainly correlated with ApoE-KO phenotype rather than any extra effect of diet. However, at 6 months, abolished OPs amplitude was observed in ApoE KO FD respect to WT ND, which represents the functional response of neuronal cells located in the INL, the most affected retinal layer by the fructose intake. These changes may be attributed to synaptic contacts loss and even neuronal death, correlating with TUNEL assay.

Regarding the microvascular alterations, ApoE-KO mice showed vascular leakage, changes in the astrocyte integrity and reduced retinal label for α-SMA accompanied by plasmatic protein extravasation at 4 months of FD. α-SMA is restricted to vascular smooth muscle cells that surround larger vessels in the normal retina (Trost et al., 2013) whereas astrocytes help to maintain vessel integrity (Zhang and Stone, 1997) and keep the barrier properties of the retinal vascular endothelium (Barber et al., 2000; Fernandez et al., 2011). Impairment of astrocytes has been reported to play a pivotal role in inner BRB breakdown, resulting in the production of vasogenic edema (Chan-Ling and Stone, 1992; Gardner et al., 1997; Rungger-Brandle et al., 2000). In the same way, an enhanced production of VEGF also underlies an increased permeability of the BRB (Kaur et al., 2008). Here, results showed Evans blue complex leakage and increased level of albumin in retinal homogenates in both WT and ApoE-KO mice fed with fructose, which suggests reduced ability to maintain BRB properties. However, despite the fact that WT and ApoE-KO FD mice showed similar vascular permeability alterations in retina the mechanisms involved in both mice groups seem to be different. Whereas in WT FD mice an increased in VEGF mRNA expression seems to be the responsible, in ApoE-KO FD mice it could be attributed to astrocyte abnormalities evidenced by reduced GFAP immunoreactivity.

Müller glial cells are the main glial cells of the retina. These cells are involved in control of angiogenesis, neurotrophic support and through their activation allow the reduction of neurotoxicity by removing metabolic waste products (Bringmann et al., 2006; Subirada et al., 2018). Despite gliosis is a feature of retinal pathology (Mizutani et al., 1998; Gardner et al., 2002), our results showed absence of GFAP reactivity in MGCs in ApoE-KO FD mice retinas at 4 months. These findings were correlated with low levels of pro-inflammatory markers such as IL-6 and TNF-α (data not shown) in whole retinal extracts indicating early stages of retinopathy.

Autophagy is a highly sensitive cellular process induced in response to a wide range of stressful conditions and its deregulation has been implicated in retinal pathologies (Boya et al., 2016; Chai et al., 2016; Amato et al., 2018). In this sense, changes in mRNA and protein levels of autophagy markers have been described in conditions of hyperglycemia and hyperlipidemia (Fu et al., 2016; Meng et al., 2017). Here, we showed that ApoE-KO FD mice retinas, in response to severe metabolic stress, were unable to increase LC3B mRNA and protein level. Confirmation of these results was obtained by immunohistochemical analysis that revealed an increased number of autophagosomes (punctate LC3B staining), mainly in the INL, in WT FD and ApoE-KO ND mice, after the CQ injection. Because of its constant recycling, LC3B transcription is not frequently increased under soft transient autophagy activation. However, when this increase persist along the time, newly synthetized protein will be necessary to support the autophagosomes formation as part of LC3BII is degraded together with the cargo. This is the effect observed in WT FD and APOE-KO ND mice, where a minimal chronic increase in LC3BII protein may require a rise in the level of transcription. All cell types rely on one or more aspects of autophagy to maintain structure and/or function in the retina (Frost et al., 2014). However, the ability to respond will depend on the status of the cell as well as the intensity and duration of the stimuli (Boya et al., 2016; Fu et al., 2016). Here, we demonstrated that retinas from WT FD mice and ApoE-KO ND mice, experienced autophagy activation and cell survival, while in mice under more severe metabolic stress as ApoE-KO FD the altered retinal function and neuronal cell death correlated with a deficient autophagy pathway activation.

Probably, the autophagy variations in ApoE-KO FD is not the mechanism underlying cell death, but the lack of activation of this pathway would be able to shift the balance to cell death in stressed cells. In this sense, it has been described several crosstalk between autophagy and apoptosis or pyroptosis, then changes in one pathway can directly or indirectly modify the cell fate (D’Arcy, 2019; Liu, 2019). Our results encourage us to keep on working in this model to unravel cellular alterations in early stages of retinopathy. Despite the small differences observed among conditions, this research showed that mice with MetS have defective autophagy activation, therefore a potential treatment could contemplate this deficit and should differ from the treatment used in hypercholesterolemic patients.

Recently, it has been shown that in rats fed with a high fructose (60%) plus high fat (9% saturated) diet, the deregulation in glucose metabolism would be the key event in the onset of weak retinal abnormalities (Vidal et al., 2019). Herein, our results clearly demonstrated that both lipid and carbohydrate derangements contributed to the early and progressive retinal impairment. In this sense, 10% of fructose in drinking water was sufficient to induce MetS as was previously reported (Wong et al., 2016). It has been reported that early visual changes caused by type 2 diabetes include color vision losses and abnormal full-field ERG (Lecleire-Collet et al., 2011; Gualtieri et al., 2013). Whereas Vidal et al. (2019) reported not changes in latencies and amplitudes of both a- and b-waves at any time, we found significant changes in the ERG response highlights a progressive reduction mainly in the OPs amplitude which strength these findings.

Taken together, our results demonstrated that ApoE-KO mice, from 2 months of FD began to present biochemical alterations representative of MetS, with deleterious consequences on retinal function, BRB permeability, as well as on intracellular recycling mechanisms. It is likely that the progression of chronic effects alter several intracellular metabolic pathways leading to complete retinal dysfunction. Therefore, these results validate ApoE-KO mice fed with FD as a suitable model of human MetS exhibiting changes associated with early signs of retinopathy, which allows to analyze the pathophysiological mechanisms involved in the progression of retinal disease as well as to develop possible therapeutic strategies in the future.

## Data Availability Statement

The raw data supporting the conclusions of this article will be made available by the authors, without undue reservation.

## Ethics Statement

All mice were handled according to guidelines of the ARVO Statement for the Use of Animals in Ophthalmic and Vision Research. Experimental procedures were designed and approved by the Institutional Animal Care and Use Committee (CICUAL) of the School of Chemical Sciences, National University of Córdoba (Res. HCD 1199/17). All efforts were made to reduce the number of animals used.

## Author Contributions

MP, PB, and MS conceived and planned the experiments. MP, PB, PS, and MR carried out the experiments. MP, PB, GC, and MS interpreted the data. GC and CC contributed to the new reagents and analytic tools, analyzed, discussed the data, and reviewed critically the manuscript. MP and MS drafted, wrote, edited, and reviewed critically the manuscript. All authors had final approval of the submitted and published versions.

## Conflict of Interest

The authors declare that the research was conducted in the absence of any commercial or financial relationships that could be construed as a potential conflict of interest.

## Abbreviations

ApoE-KO: apolipoprotein E knockout
AUC: area under the curve
BRB: blood retinal barrier
CQ: chloroquine
DM: Diabetes Mellitus
ERG: electroretinogram
FD: fructose diet
GCL: Ganglion cell layer
GFAP: glial fibrilar acid protein
GS: glutamine synthase
GSA-IB4: Griffonia Simplicifolia Isolectin-B4
HIF: hypoxia inducible factor
HDL: high-density lipoproteins
LDL: low-density lipoproteins
ILM: inner limitant membrane
INL: inner nuclear layer
IPGTT: intraperitoneal glucose tolerance test
LC3B: microtubule-associated proteins 1A/1B light chain 3B
MetS: Metabolic Syndrome
MGCs: Müller glial cells
ND: normal diet
ONL: outer nuclear layer
TUNEL: terminal deoxynucleotidyltransferase biotin dUTP nick end labeling
VEGF: vascular endothelial growth factor
VLDL: very low density lipoproteins
IDL: intermediate density lipoproteins.
